# Supplementary material for: Neural Network Training With Asymmetric Crosspoint Elements
Source: Front Artif Intell. 2022 May 9;5:891624. doi: 10.3389/frai.2022.891624 (PMC9124763; doi:10.3389/frai.2022.891624)
Supplement: Supplementary file 1 [file Data_Sheet_1.pdf]

Supplementary Materials for Neural Network Training with Asymmetric Crosspoint Elements

Authors: Murat Onen, Tayfun Gokmen, Teodor K. Todorov, Tomasz Nowicki, Jesús A. del Alamo, John Rozen, Wilfried Haensch, Seyoung Kim
Correspondence to: monen@mit.edu, tgokmen@us.ibm.com, kimseyoung@postech.ac.kr

S1.1. Theory: Derivation of the Partial Differential Equations at Continuum Limit

In this section, we first provide the equations describing the asymmetric conductance modulation model used in the manuscript (also referred to as softbound model in previous literature). Then, we derive the relation between an arbitrary intended incremental update ($\Delta G^{intended}$), and the actual resultant change in device conductance due to device asymmetry (ΔG^{real}). We incorporate that relation into the gradient descent framework and write the discrete update steps. Finally, we obtain the time evolution of the device conductance during training at continuum limit.

Exponentially saturating conductance modulation (per pulse) of devices with the characteristics shown in **Fig.1**E can be described with the following equations:

$$\Delta G^{+}(G) = \Delta G^{+}\left(G_{symmetry}\right) \times \left(1 - \kappa\left(G - G_{symmetry}\right)\right) \tag{S1}$$

$$\Delta G^{-}(G) = \Delta G^{-}\left(G_{symmetry}\right) \times \left(1 + \kappa\left(G - G_{symmetry}\right)\right) \tag{S2}$$

By definition, $\Delta G^{+}\left(G_{symmetry}\right) = -\,\Delta G^{-}\left(G_{symmetry}\right)$ and can be considered as the unit of conductance change per single pulse (consistent with the notation for a symmetric device, which is also sometimes referred as ΔG_{min} to indicate it is ultimately the resolution of the update). As a result, two equations can be combined as:

$$\Delta G^{real}(G) = \Delta G^{intended} - \kappa\left|\Delta G^{intended}\right| \times \left(G - G_{symmetry}\right) \tag{S3}$$

In Equation (S3), $\Delta G^{intended}$ is equal to $\pm\Delta G\left(G_{symmetry}\right)$. However, it can also be generalized to an approximation for integer multiples of $\pm\Delta G\left(G_{symmetry}\right)$ as well, for small enough magnitudes.

In order to capture the evolution of conductance during a training operation, we can first start investigating the discrete updates the device takes. Assuming the initial point (not to be confused with the optimal point) $G_0 = G_{symmetry}$, for a learning rate η:

$$G_1 = G_0 + \eta\Delta G_1 \tag{S4}$$

$$G_2 = G_1 + \eta \Delta G_2 - \eta\kappa |\Delta G_2| (G_1 - G_{symmetry}) \qquad (S5)$$

$$G_k = G_{k-1} + \eta \Delta G_k - \eta\kappa |\Delta G_k| (G_{k-1} - G_{symmetry}) \qquad (S6)$$

where the intended updates ΔG_k are rank-1 portions of the update matrix to be applied on the weight matrix such that

$$\sum_k \Delta G_k \propto -\eta \frac{\partial E}{\partial G} \qquad (S7)$$

Note that each ΔG is actually applied as a series of incremental updates (See S2.2), in the units of minimum conductance change of the devices. Due to asymmetry, each successive update is affected by all preceding updates (and their order). Following the notation of Equations (S4-S6), the conductance at an arbitrary instance k can be written as:

$$G_k = G_0 + \eta \left[\sum_{m=1}^{k} \Delta G_m + \sum_{n=1}^{k-1} (-\eta\kappa)^n \left(\prod_{p=k+1-n}^{k} |\Delta G_p| \right) \left(\sum_{r=1}^{k-1} \Delta G_r \right) \right] \qquad (S8)$$

It is important to realize that all ΔG_i are computed such that they correspond to the derivative of the error function with respect to G_{i-1}. Furthermore, these updates also involve a component $\epsilon(t)$, which accounts for the inherent stochasticity of this process, sampling the true gradient. Rewriting Eq.(S6) to reflect these properties:

$$G_k = G_{k-1} - \eta \left[\frac{\partial E}{\partial G_{k-1}} + \epsilon(t) \right] - \eta\kappa \left| \frac{\partial E}{\partial G_{k-1}} + \epsilon(t) \right| (G_{k-1} - G_{symmetry}) \qquad (S9)$$

which in return can be written in the continuum limit as:

$$\dot{G} = -\eta \left[\frac{\partial E}{\partial G} + \epsilon(t) \right] - \eta\kappa \left| \frac{\partial E}{\partial G} + \epsilon(t) \right| (G - G_{symmetry}) \qquad (S10)$$

as given in the main text (Equation 2).

S1.2 Theory: Derivation of Fundamental Incompatibility Between SGD and Asymmetric Devices

In this section, we will demonstrate at the equation level why asymmetric devices are fundamentally incompatible to be trained under SGD operation. We will particularly show that

the optimum point for an arbitrary optimization problem is not even stable in general for an asymmetric device modulated using SGD-computed updates.

Let us examine Equation (S10), which describes the training procedure for a single parameter optimization, as studied in Fig.1 of the main text. For the optimization process to be convergent,

$$\lim_{t \to \infty} \langle \dot{G} \rangle = 0 \qquad (S11)$$

meaning that the conductance value will settle around the vicinity of a certain level, which we here label as G_{final}. For the case of a single-parameter linear regression example, G_{final} represents the mean of the dataset.

It is important to realize that the updates over G are computed using the scalar error function E of the optimization problem. Therefore, ideally, when $G \to G_{final}$:

$$\langle \dot{G} \rangle \propto \left\langle \frac{\partial E}{\partial G} \right\rangle = 0 \qquad (S12)$$

for a successful training operation. However, for an asymmetric device, rewriting Equation (S10), at convergence yields:

$$\langle \dot{G} \rangle = -\eta \left[\left\langle \frac{\partial E}{\partial G} \right\rangle + \langle \epsilon(t) \rangle \right] - \kappa\eta \left\langle \left| \frac{\partial E}{\partial G} + \epsilon(t) \right| . \left(G - G_{symmetry} \right) \right\rangle \qquad (S13)$$

We know that $\langle \epsilon(t) \rangle = 0$ by definition, and we can substitute $\langle \dot{G} \rangle = 0$ from Equation (S12) to obtain:

$$\left\langle \frac{\partial E}{\partial G} \right\rangle = -\kappa \left\langle \left| \frac{\partial E}{\partial G} + \epsilon(t) \right| . \left(G - G_{symmetry} \right) \right\rangle \qquad (S14)$$

Equation (S14) is the analytical expression, describing the competing-forces discussed in Fig.1 in the main text. The left-hand side of the equation describes the force exerted by the optimization process, trying to set $G_{final} \to G_0$ (optimum of the problem), whereas the right-hand side is the force that pulls $G_{final} \to G_{symmetry}$. As shown in Fig.1, these two forces balance out one another to ultimately satisfy Equation (S11), but this point bears no significance for the optimization problem at hand (i.e. it does not satisfy Equation (S12))

Analytically solving Equation (S14) to find G_{final} at steady state proves to be challenging, particularly due to the unknown nature of $\epsilon(t)$ for each different case. Nonetheless, the following observations can be made:

1) The state $G_{final} = G_0$ cannot be maintained at steady state, for which $\langle \dot{G} \rangle \neq 0$, unless G_0 coincides with $G_{symmetry}$. As distance between G_o and $G_{symmetry}$ increases, G_{final} stabilizes further away from G_0 (Fig.1G).

2) Increasing magnitude of stochasticity (and circuit noise elements which are not included here for simplicity), and its distribution, strays G_{final} further away from G_0 (Fig.S1).

3) Increasing amount of asymmetry (here represented with κ) makes G_{final} stabilize further away from G_0.

As a result, asymmetric devices cannot be used to perform training tasks with SGD, as the optimal point is not even stable when the optimization starts from a "lucky-guess" of the very optimum of the problem at hand.

S1.3 Theory: Derivation of Compatibility Between SHD and Asymmetric Devices

In this section we will parallel the derivations carried out in S1.2 and demonstrate the nature of the critical difference between SHD and SGD that resolves the aforementioned stability problem.

For SHD, $A = A_{main} - A_{ref}$ gets updates computed over $C = C_{main} - C_{ref}$. Writing the evolution of A in terms of the PDE given in Equation (S10).

$$\dot{A}_{main} = -\eta_A \left[\frac{\partial E}{\partial (C_{main} - C_{ref})} + \epsilon(t) \right] - \kappa_A \eta_A \left| \frac{\partial E}{\partial (C_{main} - C_{ref})} + \epsilon(t) \right| \left(A_{main} - A_{main,symmetry} \right) \quad (S15)$$

$$\dot{A}_{ref} = 0 \quad (S16)$$

If we also write the same for C which is updated by means of partial additions of A:

$$\dot{C}_{main} = \eta_C \left(A_{main} - A_{ref} \right) - \kappa_C \eta_C \left| \left(A_{main} - A_{ref} \right) \right| \left(C_{main} - C_{main,symmetry} \right) \quad (S17)$$

$$\dot{C}_{ref} = 0 \quad (S18)$$

Note that in the actual discrete time evolution of these systems, the time-steps of $A_{main}$and C_{main} are not necessarily the same, due to the presence of τ, which is ignored here for simplicity. We again use the argument that in steady state, both A_{main} and C_{main} will converge to the vicinity of certain values ($A_{main,final}, C_{main,final}$), for which $\langle \dot{A}_{main} \rangle = \langle \dot{C}_{main} \rangle = 0$. Therefore, taking the time averages of Equations (S15) and (S17) in steady state, and substituting $\langle \epsilon(t) \rangle = 0$ yield:

$$\left\langle \frac{\partial E}{\partial (C_{main} - C_{ref})} \right\rangle = \kappa_A \left\langle \left| \frac{\partial E}{\partial (C_{main} - C_{ref})} + \epsilon(t) \right| . \left(A_{main} - A_{main,symmetry} \right) \right\rangle \qquad (S19)$$

$$\langle A_{main} \rangle - A_{ref} = \kappa_C \langle \left| \left(A_{main} - A_{ref} \right) \right| . \left(C_{main} - C_{main,symmetry} \right) \rangle \qquad (S20)$$

The ultimate goal of any optimization task is to achieve and maintain $G_{final} \approx$ at steady state, which translates to left-hand side of Equation (S19) (similar to Equation (S14)) being 0. We repeat here that for Equation (S14), this was not possible as the right-hand side was non-zero in general for that state, making the optimum point unstable. The key difference of SHD is that since A_{main} and C_{main} are different parameters, left hand side of Equation (S19) can be satisfied by $C_{main} \approx$ whereas right-hand side can be 0 when $A_{main} \approx A_{main,symmetry}$ in steady state.

On the other hand, examination of Equation (S20) reveals a critical requirement for SHD to work, which is setting $A_{ref} = A_{main,symmetry}$ (i.e. zero shifting). Under this condition and an appropriately small choice of η_A, $\langle A_{main} \rangle - A_{ref} \approx \langle \left| \left(A_{main} - A_{ref} \right) \right| \rangle \approx 0$, since A_{main} changes in the very close vicinity of $A_{main,symmetry}$ in steady state. Furthermore, $\langle A \rangle \approx 0$ also indicates that the change in $C_{main}$is negligible since the value of A is the driving force of C_{main}. This property allows us to treat $\left(C_{main} - C_{main,symmetry} \right)$ in Equation (S20) as a constant and take the term out of the averaging operator.

In addition to showing that SHD does not suffer from SGD's fundamental incompatibility with asymmetric devices, the following important observations can also be made from Equations (S15-S20):

1) The asymmetry of C_{main}, κ_C, is a less critical parameter in the training steady state as it is in multiplication form with $\langle \left| \left(A_{main} - A_{ref} \right) \right| \rangle \approx 0$.

2) The stochastic nature of the training process, $\epsilon(t)$, is filtered by the subsystem A for subsystem C due to the absolute value function behavior of device asymmetry. This observation is not immediately obvious from comparing Equations (S13) and (S17) but requires remembering that changes in C_{main} occur at a much slower rate than those of A_{main} due to the presence of τ as well as the nature of u and v (See Theory section). In short, even though we simplify equations by writing $\dot{C}_{main} \propto A$, each update on C_{main} is generated only by a fraction of information stored in A (e.g. one column for hot-encoded u choice). This statement is true even for the extreme case of $\tau = 1$. Also note that the presence of $\epsilon(t)$ in Equation (S17) is implicit within the variable A_{main}.

   In both of those equations, if all $\Delta G_{intended}$ (first term) were to be of a single sign (all positive or all negative) over a certain duration, then the correction term would simply be equivalent to a varying learning rate (dependent on G or C_{main}). Varying learning rates can alter the transient behavior by not the steady-state solution as long as they are small

enough (controlled by η or η_C). In other words, the (rapid) sign changes in the $\Delta G_{intended}$ are the core problem that interact with asymmetry, as also discussed in the main text (See Discussions).

In Equation (S13), there is nothing that prohibits rapid sign changes, as it is entirely dependent on $\epsilon(t)$ at optimum. However, for (S17), each $\Delta C_{main,intended}$ is computed over a "collection" $\epsilon(t)$ sampled by A_{main}. As a result, these different time-scales allow filtering without batching different inputs (which would require explicitly computing and storing the update matrices that cannot be done via fully-parallel operations).

3) If there is a read noise ($\lambda(t)$) when performing the forward pass $v = u.(A_{main} - A_{ref})$, then the absolute value term in Equation (S17) becomes $\left|\left(A_{main} - A_{ref} + \lambda(t)\right)\right|$. As a result, for large $\lambda(t)$, approximating this term to 0 fails, leading C_{main} to move away from the optimum. This sensitivity was indeed observed empirically in Ref. 26 for complex networks. As was also suggested by Ref. 26, countermeasures can be implemented via Schmidt trigger-alike" elements, or other similar hysteretic techniques.

The result of all this is that asymmetric devices can be used in optimization tasks under SHD-based training.

S2.1. Methods: Performing Parallel Matrix-Matrix Multiplication with Crossbar Architectures ($y = Wx$ and $z = W^T\delta$):

In this section we discuss how to execute fully-parallel matrix-matrix multiplication using crossbar architectures. The example will be given for forward pass ($y = Wx$). For backward pass ($z = W^T\delta$), the operation is the same, except for all rows and columns are interchanged.

- Input vector (x) is represented as time-encoded voltage pulses (i.e. higher value → longer pulse).

- Voltage pulses are applied to the row terminals of array W (and inverted version to those of the reference array). Column terminals are connected to operational amplifiers (in integrator configuration), which are at virtual ground potential.

- Each crosspoint element (at row i, column j) provides charge ($Q_{i,j}$) to the operational amplifiers, proportional to the integral of the product of crosspoint conductance ($G_{i,j}$), and row voltage (V_i) (i.e. $Q_{i,j} = \int G_{i,j}.V_i dt$) over a predetermined integration time (t_{int}).

- At the end of each column, integrators accumulate the line currents (Kirchhoff's Law), such that the output voltage level (V_j) is proportional to the vector (x) matrix (W) inner product (i.e. $V_j \propto \sum_i \int G_{ij} V_i dt$).

- Analog voltage level is converted into digital using an analog-to-digital converter (ADC). The digital output vector is further processed as required (e.g. activation function, pooling), and stored in digital domain.

S2.2 Methods: Performing Parallel Update with Crossbar Architectures ($W \leftarrow W - \eta . x \otimes \delta$):

In this section we discuss how to execute fully-parallel matrix update on crossbar architectures.

- Update cycle is done via a stream of pulses, each modulating device conductance incrementally. Expected crosspoint behavior is that the conductance change only occurs when the potential across the update terminals are greater than a threshold magnitude (${V_{th}}^{update}$).

- For each layer (W), the corresponding input (x) and error (δ) matrices are obtained from the memory. Dimensionally, if W is an $m \times n$ matrix, x would be an $m \times p$ matrix, and δ would be a $p \times n$ matrix. The update computation and application for these matrix dimensions are performed by p Rank-1 updates.

- For each row of x (and column of δ), every value in those vectors are represented with a stream of pulses. These pulses have binary logic characteristics, such that they are either populated (i.e. there is a pulse of certain voltage level, "1"), or not ("0"). The voltage levels for "1" pulses are selected in regard to the ${V_{th}}^{update}$ value of crosspoint devices, such that those for x vector attain $+\frac{{V_{th}}^{update}}{2}$, whereas for δ $-\frac{{V_{th}}^{update}}{2}$ is used.

- Here we will cover the stochastic pulse generation, while deterministic alternatives also exist for a parallel operation. In this setting, the values in x and δ determine the probability of population for the slots of the pulse stream (i.e. whether or not a pulse is generated, "1", or not "0"). Total number of pulse positions is often referred as Bit Length (BL). Higher the value (in magnitude), more pulse positions will be populated (i.e. a pulse will be sent).

- Pulse streams corresponding to x are sent to the crossbar from row-update terminals, while those of δ are sent from column-update terminals at the same time. As a result, the crosspoint element with index i, j will have a potential difference across its update terminals of: 0, $+\frac{{V_{th}}^{update}}{2}$, or $+{V_{th}}^{update}$, depending on the pulses generated at that instance from row i and column j. As designed, from these options, only $+{V_{th}}^{update}$ will actuate an incremental conductance change ($+dG$), which corresponds to the ["1","1"] state for the pulses generated at that instance from row i and column j.

- As the probability of "1" state at a row pulse position i is proportional to the value of x_i, and that of column j, is proportional to the value of δ_j, the probability of two pulses coincide (i.e. $[1,1]$ state) on the crosspoint element with indices i, j is proportional to the product of $x_i . \delta_j$.

This logic ensures that the entire array to get updated together proportional to $x \otimes \delta$, without computing the outer product explicitly.

- The steps described above only allows crosspoint conductance to change in positive direction. To support negative changes, all update cycles are performed in 4 subcycles which are summarized in the table below.

x values	δ values	Row "1" Voltage	Column "1" Voltage	["1", "1"] State	Conductance Change
+	+	$+\frac{V_{th}^{update}}{2}$	$-\frac{V_{th}^{update}}{2}$	$+V_{th}^{update}$	$+dG$
+	-	$-\frac{V_{th}^{update}}{2}$	$+\frac{V_{th}^{update}}{2}$	$-V_{th}^{update}$	$-dG$
-	+	$-\frac{V_{th}^{update}}{2}$	$+\frac{V_{th}^{update}}{2}$	$-V_{th}^{update}$	$-dG$
-	-	$+\frac{V_{th}^{update}}{2}$	$-\frac{V_{th}^{update}}{2}$	$+V_{th}^{update}$	$+dG$

Note that although it seems like the algorithm would require infinitely long pulse-streams ($BL \to \infty$) to accurately compute the outer product, we have empirically found that optimal values for $BL \approx 5 - 20$. For more detailed analysis of this method, we refer the reader to Refs. (*9*, *10*).

S2.3 Methods: Simulation Details for Figures 1 and 2

The simulated results shown in Figures 1 and 2 are based on the device models depicted in Figure 1 (also known as the softbound model). These models rely on the asymmetric modulation behavior given in Equations (S1-S3).

A dataset is synthetically created for a single parameter linear regression problem in the form of $y = G_0 x_1 + \gamma$ where G_0 are the unknowns searched for and γ is the Gaussian noise. Training is performed using a sum of squared error cost function in both SGD and SHD cases.

S2.4 Methods: Experiment Details For Figure 3

The device technology used in the demonstration of SHD algorithm is metal-oxide based electrochemical random-access memory (MO-ECRAM). Material choice and fabrication flow of these devices are CMOS- and BEOL-friendly, which is a major improvement with respect to their Li-ion based predecessors. These 3-terminal devices have reportedly shown non-linear gate I-V characteristics, which enables programming without selector or access devices in an array.

Electrical characterization setup consists of Tektronix 70404C mixed signal oscilloscope, Agilent 81150 arbitrary pulse generator, Keithley 2400 SMU, and a Keithley 707B switch mainframe.

Training was performed using a synthetic dataset of 100 $x_{1,2}$ and y generated of form $y = G_{0_1}x_1 + G_{0_2}x_2 + \gamma$, where $G_{0_{1,2}}$ are the unknowns searched for and γ is the Gaussian noise. Input values $x_{1,2}$ had a mean of 0 and a standard deviation of 1. Differential conductance values that correspond to $G_{0_{1,2}}$ were 3.5μS and $= -6.5\mu S$ respectively. Symmetry points for $A_{main,1,2}$ were found to be $1.07\mu S$ and $35.4\mu S$, which were recorded at the beginning of the training operation as A_{ref} (also used as C_{ref}). The high variation in the symmetry points were due to the different physical device sizes used, in order to find 4 operational devices across the fabricated chip. 10 pulses were generated at each update (i.e. $BL = 10$), where τ was selected to be 10.

On the retrospect, we found that the high amount of residual motion in all parameters at steady state (Fig. 3D), was due to the selection of few input values (100 $x_{1,2}$). Instead, a larger dataset, created with same statistical variations produce much cleaner steady state behavior, which is also the case for the LSTM simulations shown in Fig4.

<u>S3.1 Additional Results: LSTM Simulation for PCM-like Devices</u>

We have performed the same training operation for devices with models based on PCM-like behavior. The most important aspect of these devices is the abrupt resetting characteristics as shown in the inset of **Fig. S3**A. As a result, such devices do not have a well-defined symmetry point and thus are not applicable for training with SHD algorithm. **Fig. S3** verifies these assertations as neither SGD not SHD can achieve successful training results when all operations are kept fully-parallel

<u>S3.2 Additional Discussions: Plausible Misinterpretation of SHD Operation</u>

One possible misinterpretation of the SHD method is to perceive A_{main} as the subsystem that simply integrates the updates on a "quasi-symmetric regime" (i.e. in the vicinity of its symmetry point where the asymmetry is the lowest), which is then passed to C_{main}. This would form a counter-like open-loop system, and there are indeed approaches that work in a similar manner (Ref. 14). For SHD, if A_{main} was indeed simply accumulating information in a quasi-symmetric range of values, then a symmetric device would be perfectly suitable (since any range would satisfy this requirement). However, it can be seen from Fig.2E and Fig.4B that it is not the case.

SHD does not try to comply with asymmetry, or operate where asymmetry is least pronounced, but on the contrary, it relies on asymmetric modulation. This is best shown by the physical analogy we discuss. Investigating the PDEs given in Fig.2C and Fig.2D, it can be seen that the asymmetry (on A_{main}) corresponds to a drag-like force; that introduces dissipation to the system. In the absence of such dissipation, the system oscillates indefinitely. Indeed, for this 1-D problem, it can be seen in Fig.2E, the parameter that is optimized (with a symmetric device) is not convergent under SHD operation.

Naturally, any DNN operation is much more complex than this 1-D problem. As a result, the learning curves do not simply display oscillations as in the case of Fig.2E. However, the inherent incompatibility between SHD and symmetric devices yield the results shown in Fig. 4B. It is important to note that this model corresponds to a "perfectly symmetric" device, whereas the slightest degree of asymmetry (shown with the blue curve in the inset of Fig.4A) not only works well, but outperforms "perfectly symmetric" device trained with SGD by means of both conversion speed and final accuracy.

S3.3 Additional Discussions: Momentum-SGD Based Solutions

Examining the source of problem, one can realize that a method to reduce the effect of $\epsilon(t)$ would be to adopt a momentum-based method (e.g. Momentum-SGD, Adam, AdaGrad, RMSProp). Such a method would certainly ameliorate the issue, as it would strengthen the gradient, but it cannot be implemented in a parallel fashion.

Momentum SGD requires previous update matrices in the computation of the next update matrix. This indicates that all update matrices are computed and stored explicitly (the outer product operation is executed in digital domain). Instead, the update method we use computes and applies those update matrices, without returning the result of the outer product to the user. Therefore, it cannot be stored for next update matrices for applying a gradient method. Furthermore, even if one were to read the entire matrix before and after to generate the update matrix post-facto, it would still not be enough to use a momentum method with parallel operations, as there are element-wise operations required for the momentum SGD.
The only possible way to have a momentum method while remaining parallel is to use crosspoint devices that inherently show such characteristics. We do not find this feasible either as in momentum SGD, the decay factor is a critical hyperparameter that requires tuning for different training scenarios.

S3.4 Additional Discussions: Simulation Choice and Limitations

It is important to realize that SHD only resolves asymmetry related issues. Therefore, other imperfections related with analog processors such as device-to-device variability, cycle-to-cycle variability, noise, and resolution can still deteriorate the training performance significantly. These effects are empirically known to be further pronounced for more complex networks and datasets.
Large-scale problems such as training ResNet20 on CIFAR-10 dataset and AlexNet on ImageNet dataset are studied in the work 'Training Large-scale Artificial Neural Networks on Simulated Resistive Crossbar Arrays' by M.Rasch, T. Gokmen and W. Haensch in 2019. In that manuscript, authors found that simulating asymmetric devices for those networks resulted in very poor results, and consequently chose to study other imperfections instead. Even then, they report : "We find that using AlexNet off-the-shelf is not trainable with our baseline RPU model

(even with floating point update but limited ADC/DAC resolution)”[1]. In other words, even with perfectly symmetric devices (trained with SGD), analog arrays could not generate FP32-level training performance due to other nonidealities. Given that SHD only resolves asymmetry, we chose not to replicate the results in this aforementioned reference.

Furthermore, to give a reference point, the LSTM simulations we provide take ~6 hours each on IBM’s cluster. Moreover, those simulations are not singular-runs but belong to a set of simulations where hyperparameters (such as η_A, η_C, and τ) are cross-swept for each device type (i.e. different asymmetry levels and models).

On the other hand, the LSTM problem we study in detail is an optimal problem, which is complex enough to validate the training algorithm, while it still is trainable with limited number of conductance states, analog noise, variations, and limited resolution. We recommend future studies to explore larger problems, once there are additional solutions for these other nonidealities related to analog crossbar architectures.

S3.5 Additional Discussions: Asymmetry Related Performance Degradation as a Function of Standard Deviation of the Dataset

As described in both the Theory and the Discussions sections, the level at which device asymmetry interferes with the optimization process is dependent on the amount of statistical variation present in the training dataset. For any practical application, this is not a parameter that the user can adjust. However, for demonstrational purposes we revisit our single-parameter linear regression example, where we can adjust the standard variation of the input data for which the mean is intended to be computed. **Fig.S1** shows Euclidean norm between the final convergence point and the optimal point, with respect to an increasing level of standard deviation in the dataset. It can be seen that for symmetric devices, higher dataset variation does not create any penalty in the optimization result as expected. However, when the devices are asymmetric, the level of statistical variation determines the degree at which asymmetry is “picked-up”. On the other hand, this is again not an issue when those asymmetric devices are trained with SHD algorithm as this method can efficiently mitigate the degrading effects of device asymmetry.

S3.6 Additional Discussions: 3-Array Operation

In order to verify the idea to discard C_{ref} and use A_{ref} as a common reference array, we have performed simulations for all neural networks studied in Ref. (*26*). For illustration purposes, here we only present the learning curves obtained for a convolutional neural network example. **Fig.S2**A shows simulated learning curves for configurations with a discrepancy introduced between A_{ref} and $A_{symmetry}$, and **Fig.S2**B shows the same for C_{ref} and $C_{symmetry}$. The details of the simulation framework can be found in Ref. (*10*). In simulations, weight bounds

[1] RPU stands for Resistive Processing Unit, which is another name for crossbar array with analog devices. Floating point update means, there is no device asymmetry, and the outer products are computed and applied in an exact fashion.

(maximum absolute value of a weight) have a mean value of 0.6. Standard deviation levels, σ can be best understood in relation to this value (i.e. the weight range). The $\sigma = 0$ case refers to correct initialization of the reference array, such that $W = W_{main} - W_{ref} = 0$ when $W_{main} = W_{main,symmetry}$. For any other value of σ, $W \neq 0$ at those conditions, but instead is a random matrix with a mean proportional to σ.

It was observed that the training accuracy is strongly dependent on proper initialization of A_{ref} in agreement with the analyses carried out in the Theory section, whereas the subsystem C does not show such sensitivity. Therefore, utilizing 3 asymmetric crossbar arrays per layer is sufficient to implement the SHD algorithm, which provides classification accuracy equivalent to that obtained for SGD-based training with ideal devices.

In all analyses, we assume that conductance values of $A_{main,symmetry}$ exist in A_{ref}. This assumption needs to hold for both 3 and 4 array operation. The only remaining requirement after that is those values of $A_{main,symmetry}$ also lie in the mid-range of C_{main} as well, such that the values of $[C_{main} - A_{ref}]$ (which is equal to $[C_{main} - A_{main,symmetry}]$) can take roughly the same range of positive and negative numbers. Here it is worthwhile to state that we expect all arrays to be produced the same way (i.e. belonging to the same statistical distribution), and can be interchangeable.

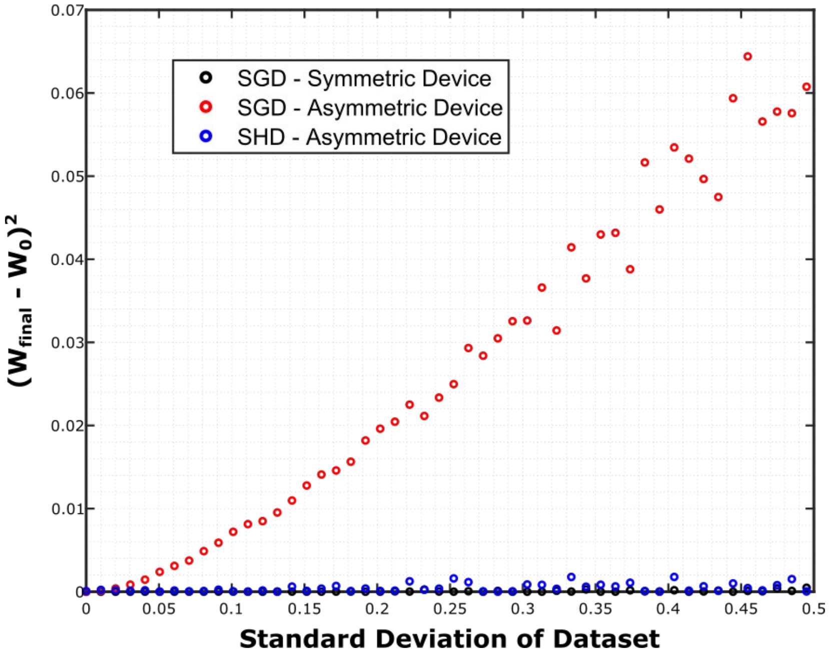

Fig. S1.

Resultant optimization error as a function of standard deviation of the input data for the single-parameter optimization example discussed in Fig.1.

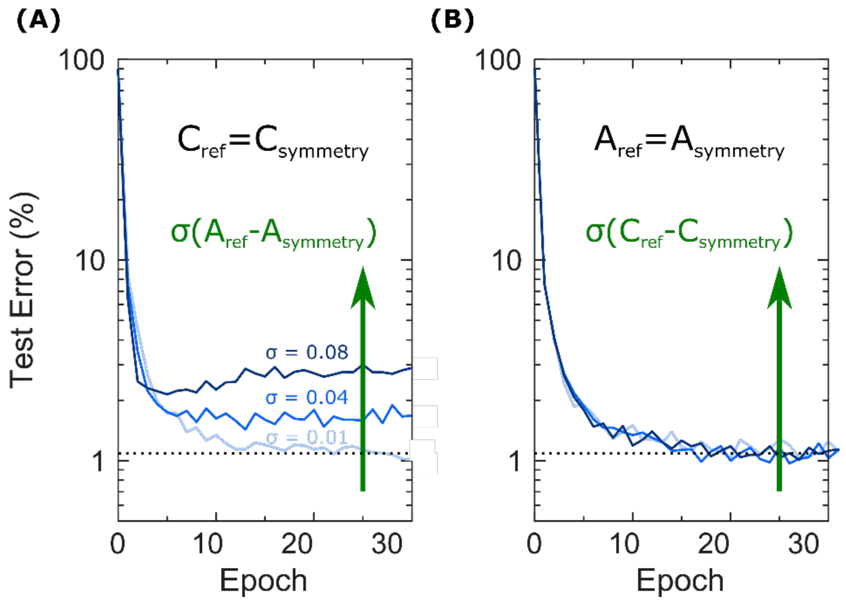

Fig. S2.

Simulated training results with SHD algorithm for a convolutional neural network with ≈8×10^5 weights on MNIST dataset for modeled asymmetric device characteristics. (A) The learning curve for different levels (i.e. standard deviation, σ) of discrepancy introduced between A_ref and A_symmetry, while C_ref is accurately initialized to C_symmetry. (B) The learning curve for different levels of discrepancy introduced between C_ref and C_symmetry, while A_ref is accurately initialized to A_symmetry. The average baseline error (only the final value) for ideal devices with conventional SGD is shown with the dashed line. Network details can be found in Ref.(10).

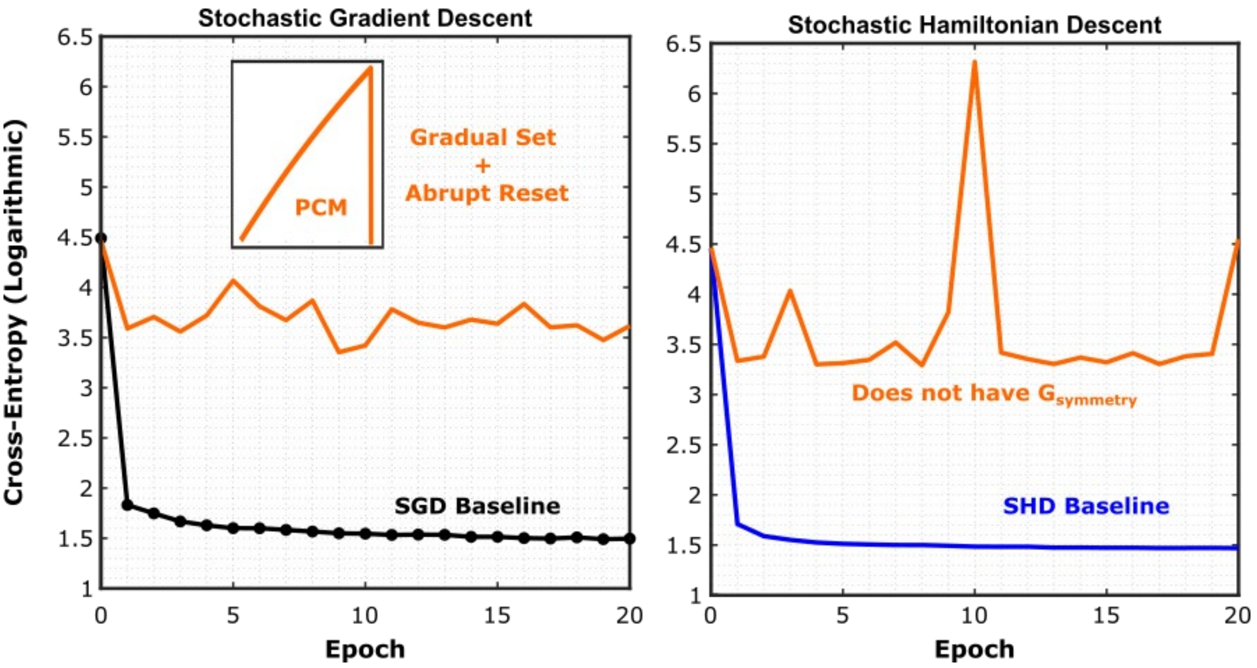

Fig. S3.

Simulated training results for PCM-like devices. (A) Simulated learning curves of the LSTM network shown in Fig.4 using PCM device models under the SGD algorithm. Conductance modulation characteristics for a typical PCM device is given in the inset. SGD baseline corresponds to training with perfectly symmetric device. (B) Simulated learning curves of the same network using PCM device models under the SHD algorithm. SHD baseline corresponds to training with device model labeled as “low” asymmetry.

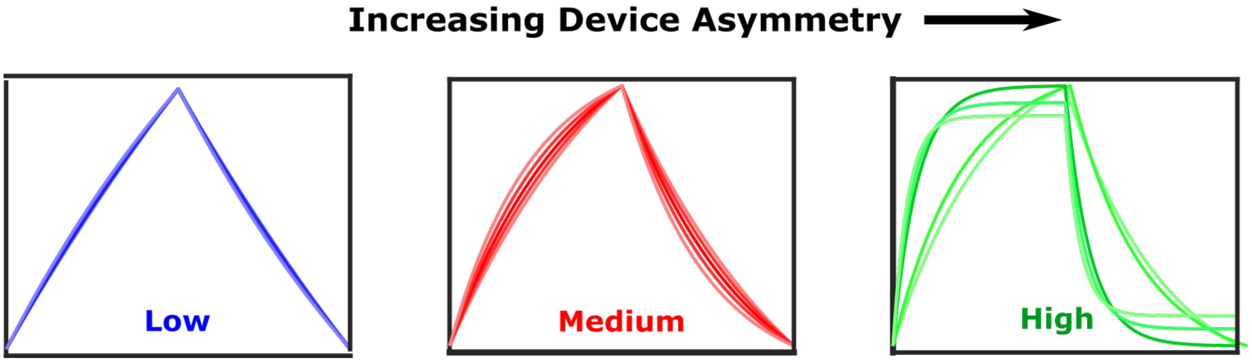

Fig. S4

Conductance modulation characteristics of different device models used in LSTM simulations shown in Fig.4, accounting for the device-to-device variation and cycle-to-cycle variation. In training simulations, 30% of cycle-to-cycle variation was used whereas device-to-device variation was selected to be 10% across all arrays.

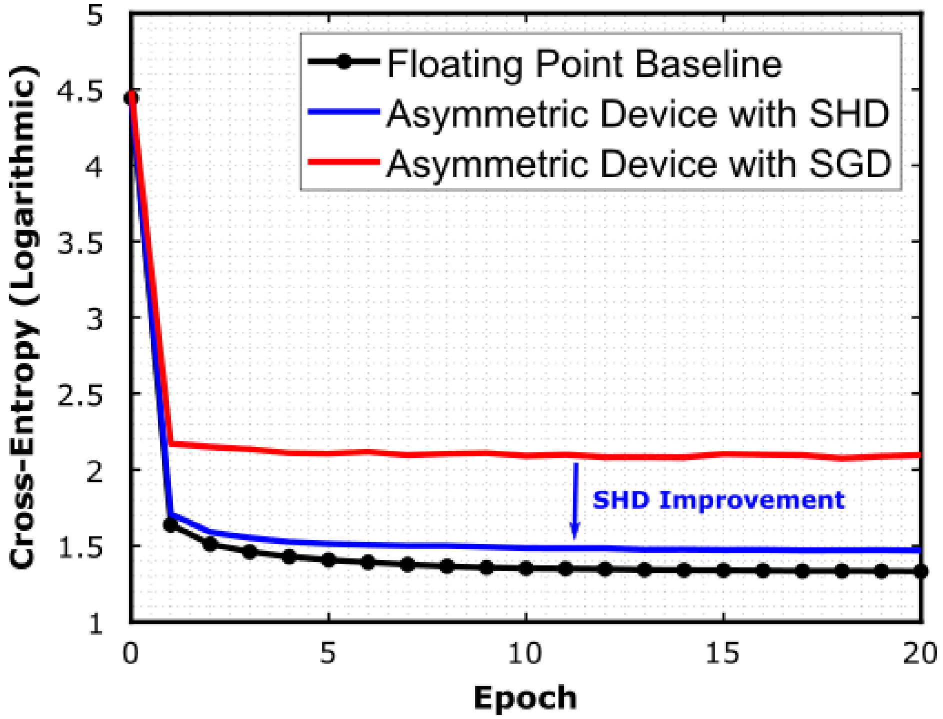

Fig.S5.

Simulated learning curves for an LSTM network trained on War and Peace dataset shown in Fig.4, showing the floating point (i.e. digital) baseline of 1.3315 cross-entropy score with respect to 1.490 of SHD and 2.0960 of SGD.
